# TiO_2_ Nanohelices Decorated with Homogeneous Au‐Core Pd‐Shell Nanocatalysts for Selective Toluene Gas Detection

**DOI:** 10.1002/smll.202504976

**Published:** 2025-07-06

**Authors:** Hyeonwoong Hwang, Hanseo Bae, Eunji Ahn, Dongmin Lee, Hyeon Ho Cho, Sunah Cheong, Tae Yeon Kim, Jaerim Kim, Yongju Yun, Donghwa Lee, Sei Kwang Hahn, Jong Kyu Kim

**Affiliations:** ^1^ Department of Materials Science and Engineering POSTECH Pohang 37673 Republic of Korea; ^2^ Division of Advanced Materials Science POSTECH Pohang 37673 Republic of Korea; ^3^ Department of Chemical Engineering POSTECH Pohang 37673 Republic of Korea

**Keywords:** bimetallic nanocatalyst, density functional theory, homogeneous decoration, titanium dioxide, toluene gas sensor

## Abstract

The precise decoration of bimetallic nanocrystals (NCs) with uniform size and homogeneous composition on metal oxide (MOX) surfaces is crucial for developing highly sensitive and selective MOX‐based gas sensors. In this study, MOX‐based gas sensors are present decorated with homogeneous Au‐Pd bimetallic (Au@Pd) NCs synthesized via seed‐mediated sequential reduction of Au and Pd on an array of TiO_2_ nanohelices (NHs) matrix. Due to the uniform composition, size, and dispersion of the bimetallic NCs, the sensor exhibits outstanding toluene (C_7_H_8_) sensing performance. The optimized Au@Pd NC composition (Au:Pd = 55:45) facilitates chemisorbed oxygen spillover and electronic sensitization, achieving an exceptionally high response (R_a_/R_g_) of ≈130 000 and rapid response/recovery (69 s/4 s) toward 100 ppm of C_7_H_8_ at 200 °C. Furthermore, the homogeneity of Au@Pd NCs enhances selectivity by providing controlled active sites, yielding a 1008‐fold higher response to toluene compared to acetone. Density functional theory calculations and mechanistic experiments reveal that Au@Pd NCs generate toluene‐selective catalytic sites that enable complete oxidation. The findings demonstrate that homogeneous core‐shell bimetallic NCs can be uniformly integrated on a highly porous MOXs‐based gas sensing matrix, enabling exceptional selectivity and sensitivity for advanced gas sensor applications.

## Graphical abstract

Homogeneous composition of Au@Pd nanocatalysts decorated on TiO_2_ nanohelices enable ppb‐level toluene detection with exceptional selectivity. Au@Pd nanocatalysts serve as critical active sites for the complete and rapid oxidation of toluene, and the site‐specific toluene oxidation mechanism is unveiled through synergistic DFT modeling and in situ DRIFTS validation.

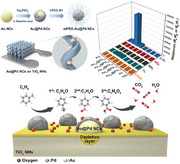

## 1 Introduction

Toluene, a colorless and aromatic volatile organic compound, is widely used in commercial products such as adhesives, inks, paints, rubber cements, detergents, pharmaceuticals, and preservatives.^[^ ^1^ ^]^ Prolonged exposure to toluene can severely affect the nervous system, leading to disturbances in brain function, balance, vision, hearing, and speech.^[^ ^2^ ^]^ Moreover, toluene is regarded as a distinct cancer biomarker as elevated levels have been detected in the exhaled breath of lung cancer patients.^[^ ^3^, ^4^, ^5^ ^]^ Therefore, a gas sensor capable of detecting trace levels of toluene is essential for preventing neural malfunction and enabling the non‐invasive diagnosis of lung cancer.

Semiconducting metal oxides (MOXs)‐based chemiresistive gas sensors are one of the promising devices form detecting trace levels of toluene.^[^ ^6^ ^]^ Their operation relies on resistance changes induced by reactions between gaseous molecules and chemisorbed oxygen species on the surface of the MOXs including TiO_2_, SnO_2_, WO_3_, and ZnO.^[^ ^7^, ^8^, ^9^, ^10^, ^11^, ^12^, ^13^ ^]^ Incorporating noble metals (Au, Pt, Pd, Rh, etc.) into MOX materials can significantly enhance sensing performance by leveraging catalytic effects and oxygen spillover.^[^ ^14^, ^15^, ^16^, ^17^, ^18^ ^]^ Moreover, bimetallic nanocatalysts (NCs) decoration offers a synergistic effects and tunable catalytic properties, dictated by composition and atomic arrangement of each metal.^[^ ^19^ ^]^ Since chemical reactions with gaseous molecules occur preferentially at active surface sites, precisely engineering the composition and structure of bimetallic NCs is imperative for superior catalytic performance.

Recently, extensive attempts have been made to explore novel material combinations and unique bimetallic NC compositions.^[^ ^20^, ^21^, ^22^ ^]^ Gaurav Pandey et al. synthesized AuPd alloy NCs decorated SnO_2_ via the solution based one‐pot reduction method, achieving high response at low temperature (175 °C) for hydrogen detection.^[^ ^23^ ^]^ Liu et al. decorated bimetallic AuPd NCs on WO_3_ nanowire bundles by using solution‐based sequential reduction method, showing fast response/recovery toward n‐butanol gas at 200 °C.^[^ ^24^ ^]^ Despite of the advantages of bimetallic NCs decoration, the selectivity of the gas sensors remains a challenge, mainly due to heterogeneous bimetallic NC compositions, which hinder the design of highly selective and sensitive MOXs‐based gas sensors.

In this study, we introduce a rational two‐step fabrication process comprising the synthesis of homogeneous bimetallic NCs and their controlled decoration onto a nanostructured MOXs gas sensor. Composition‐controlled, and uniformly dispersed Au@Pd NCs conjugated with Methyl polyethylene glycols‐thiol (mPEG‐SH) were synthesized. These NCs were dispersed and calcined on an array of TiO_2_ nanohelices (NHs) to form unique Au core‐Pd shell (Au@Pd) structure. The Au@Pd NCs‐decorated gas sensor with an optimized composition of Au:Pd = 55:45 (Au_55_Pd_45_) demonstrates exceptional sensitivity and selectivity toward toluene gas. The high sensitivity is attributed to oxygen spillover and electrical sensitization effects induced by bimetallic Au@Pd NCs, while the outstanding selectivity results from their homogeneous composition and unique core‐shell structure. Density functional theory (DFT) calculations further reveal the origin of the toluene selectivity in the homogeneous Au core‐Pd shell structure, and the toluene gas sensing mechanism was experimentally explored via in situ diffuse reflectance infrared Fourier transform spectroscopy (DRIFTS) measurements. This strategy can be extended to various material combinations, paving the way for the design of homogeneous bimetallic NC‐incorporated MOX‐based gas sensors with unparalleled selectivity for specific gases.

## 2 Results and Discussion

### 2.1 Fabrication of Homogeneous Au@Pd NCs Decorated TiO_2_ NHs Gas Sensor

To fabricate a toluene gas sensor decorated with homogeneous bimetallic Au@Pd NCs, a two‐step process consisting of NC synthesis and NC decoration. This separation of fabrication steps effectively minimizes the degree of disorder, which can arise from simultaneous mixing of MOX and bimetallic NC precursors. By focusing solely on the synthesis steps, we achieved precise control over the composition and size of the Au@Pd NCs, ensuring greater homogeneity when incorporated into the TiO_2_ NHs.

**Figure 1:**
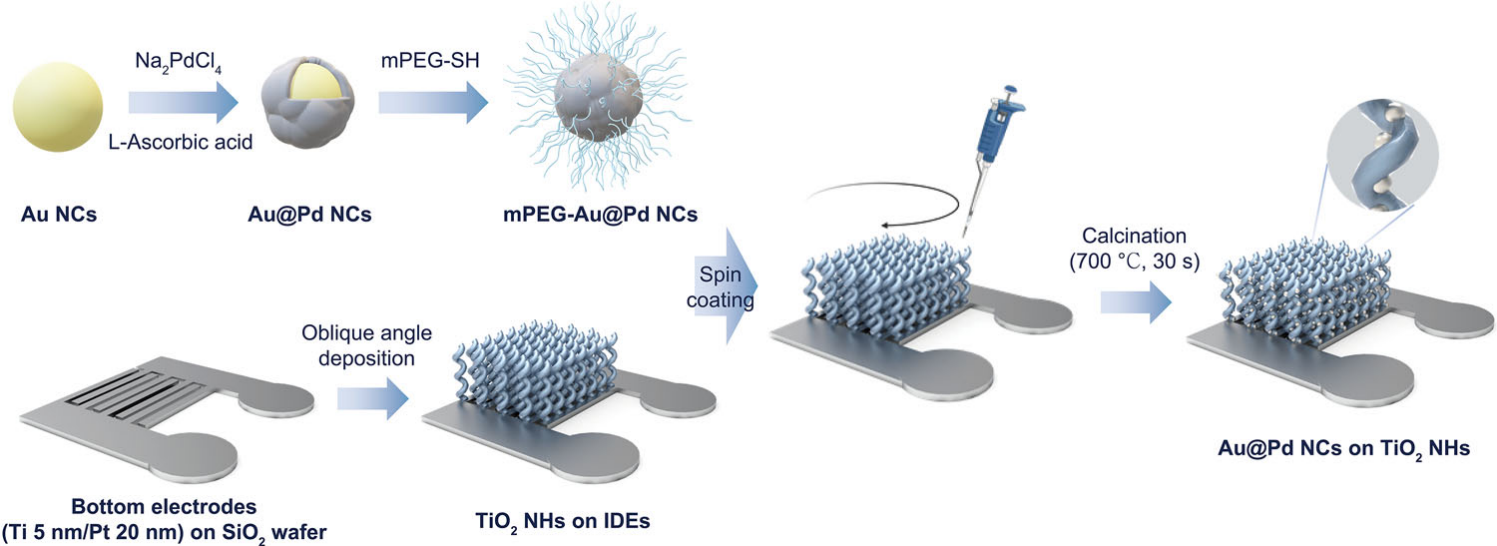
Schematic illustration of fabrication procedure for a homogeneous Au@Pd NCs decorated TiO_2_ NHs gas sensor.

The entire synthesis steps of methoxy polyethylene glycol‐thiol (mPEG‐SH) conjugated Au@Pd NCs (mPEG‐Au@Pd NCs) and their decoration on TiO_2_ NHs are illustrated in **Figure** **1**. To form the core of the bimetallic Au@Pd NCs, Au NCs of uniform size were synthesized by adding gold (III) chloride hydrate (HAuCl_4_) to a boiling solution of trisodium citrate (Na_3_Ct) dissolved in deionized (DI) water, followed by filtering the aggregated nanoparticles with a poly vinylidene fluoride (PVDF) syringe filter. Subsequently, to grow Pd nanoclusters on the surface of Au NCs, sodium tetrachloropalladate (II) (Na_2_PdCl_4_) and L‐ascorbic acid were added to the Au NCs seed solution and stirred at room temperature. Since the nucleation of the Pd nanoclusters on the Au NCs surface is thermodynamically favorable, Pd preferentially grows on the surface of spherical Au NCs rather than forming independent Pd NCs.^[^ ^25^ ^]^ This process enhances the homogeneity of the bimetallic NCs. The prepared solution was filtered again using a PVDF filter to ensure uniform NC size. Finally, mPEG‐SH (molecular weight = 10 KDa) solution was added to functionalize the Au@Pd NCs, preventing agglomeration.

Along with the NCs synthesis, an array of TiO_2_ NHs was deposited onto Ti/Pt (5 nm/20 nm) interdigitated electrodes (IDEs) employing the oblique angle deposition (OAD) method to form the gas sensing layer. In the OAD process, the vapor flux of the source material is incident at a specific angle (𝜃) with respect to the normal direction of the substrate. During the initial deposition stage, nano‐sized nuclei are randomly formed on the substrate, creating self‐shadowed regions that the vapor flux cannot reach. Subsequently, the vapor flux preferentially deposits on top of these nuclei, leading to the formation of highly porous and vertically aligned nanostructures. These structural features offer excellent accessibility for both the mPEG‐Au@Pd NCs solution and reaction gases. In the final step, the mPEG‐Au@Pd NCs solution was spin‐coated onto the array of TiO_2_ NHs for uniform dispersion. After drying DI water‐based solvent, rapid calcination was carried out at 700 °C for 30 s to thermally decompose mPEG and securely immobilize the Au@Pd NCs onto TiO_2_ NHs.

### 2.2 Structural and Chemical Characterization of Au@Pd NCs Solution and Au@Pd NCs Decorated TiO_2_ NHs

**Figure 2:**
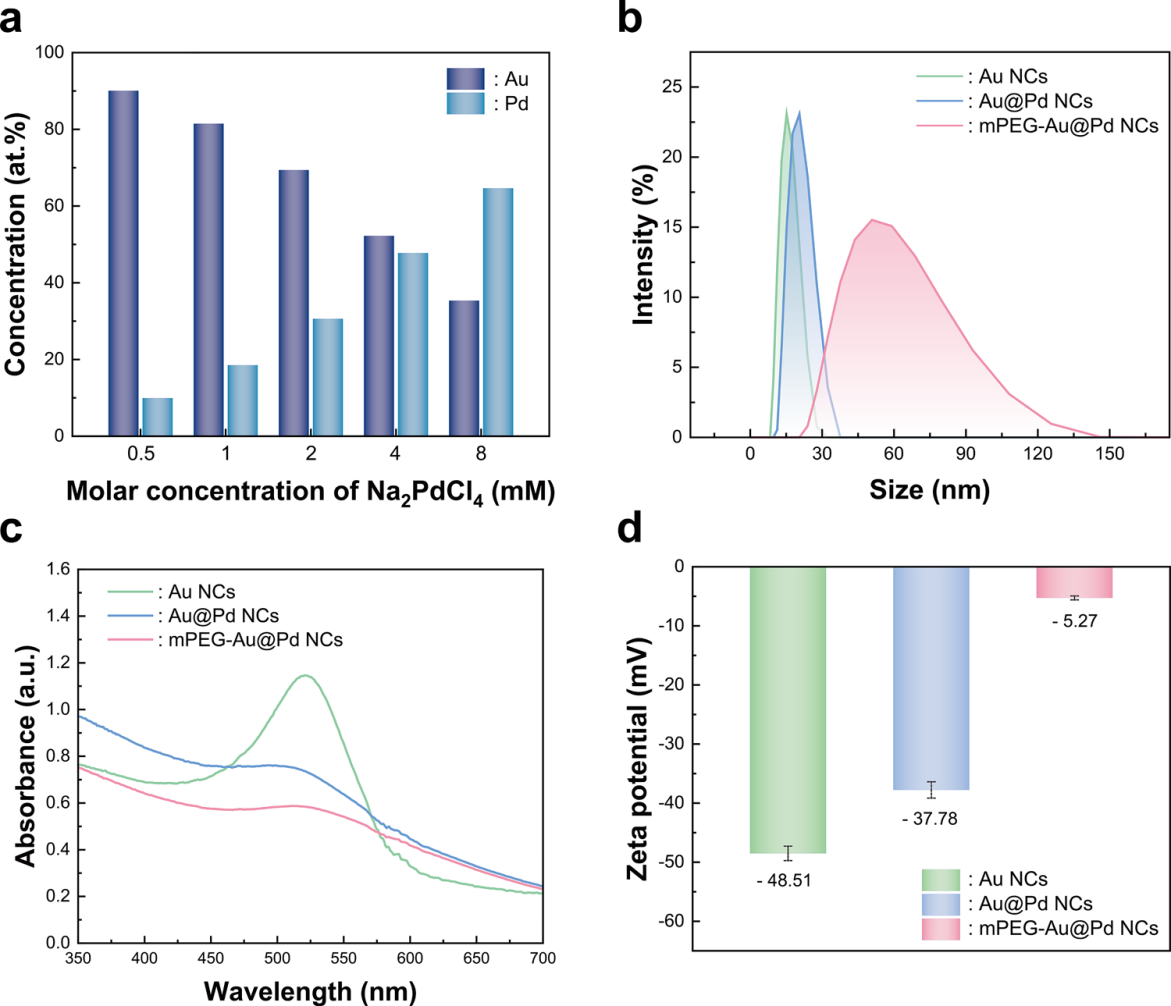
a) The atomic ratio of Au and Pd in the mPEG‐Au@Pd NCs varies with Na_2_PdCl_4_ concentration, measured by ICP‐OES. b) The hydrodynamic particle size by DLS analysis, c) the UV–vis spectra, and d) the zeta potential results (n = 5) of the synthesized Au NCs, Au@Pd NCs and mPEG‐Au@Pd NCs.

To understand the effectiveness of the fabrication methods, bimetallic Au@Pd NCs were characterized in both their as‐synthesized solution state and after decoration onto TiO_2_ NHs. **Figure** **2**a shows the concentration ratio of each metal in the as‐synthesized Au@Pd NCs, estimated by using inductive coupled plasma optical emission spectroscopy (ICP‐OES), gradually changes with the amount of Pd precursor. The Au@Pd NCs produced with 4 mM Na_2_PdCl_4_ resulted in a composition ratio of Au:Pd = 55:45, and showed outstanding gas sensing performance. This sample is referred to as Au_55_Pd_45_ and will represent the group of bimetallic Au@Pd NCs unless otherwise specified.

Figure 2b–d shows the characterization of the NCs at each synthesis step using dynamic light scattering (DLS) analysis, UV–vis spectroscopy, and zeta potential measurements. The DLS spectra show that addition of Pd and mPEG to the surface of Au NCs results in an increase in average particle size from 15.1 to 19.4 nm and 49.6 nm, respectively. The uniform size distribution is supported by the low polydispersity index (PDI) value of 0.15 for the mPEG‐Au@Pd NCs. The UV–vis spectra further demonstrate the incorporation of Pd and mPEG onto the Au NCs surface during the synthesis process. The absorbance peak of Au NCs at ≈520 nm corresponds to their surface plasmon resonance (SPR) peak. This peak shifts toward shorter wavelengths upon the growth of Pd on the surface of the Au NCs, indicating the formation of the Au@Pd NCs. The shift and weakening of the SPR peak observed after the formation of the bimetallic structure can be attributed to changes in the electronic structure induced by the addition of Pd.^[^ ^26^ ^]^ The spectrum of mPEG‐Au@Pd NCs becomes broader and red‐shifted compared to that of the Au@Pd NCs, indicating an increased particle size.^[^ ^27^ ^]^ The zeta potential shifted from −48.51 mV for citrate‐capped Au NCs to −5.27 mV for mPEG‐Au@Pd NCs. The increase in the zeta potential values proves that the bimetallic NCs were successfully coated with mPEG.^[^ ^28^ ^]^ The PEGylation layer of mPEG performs a role in increasing steric hindrance and contributes to prevent aggregation of the Au@Pd NCs.

**Figure 3:**
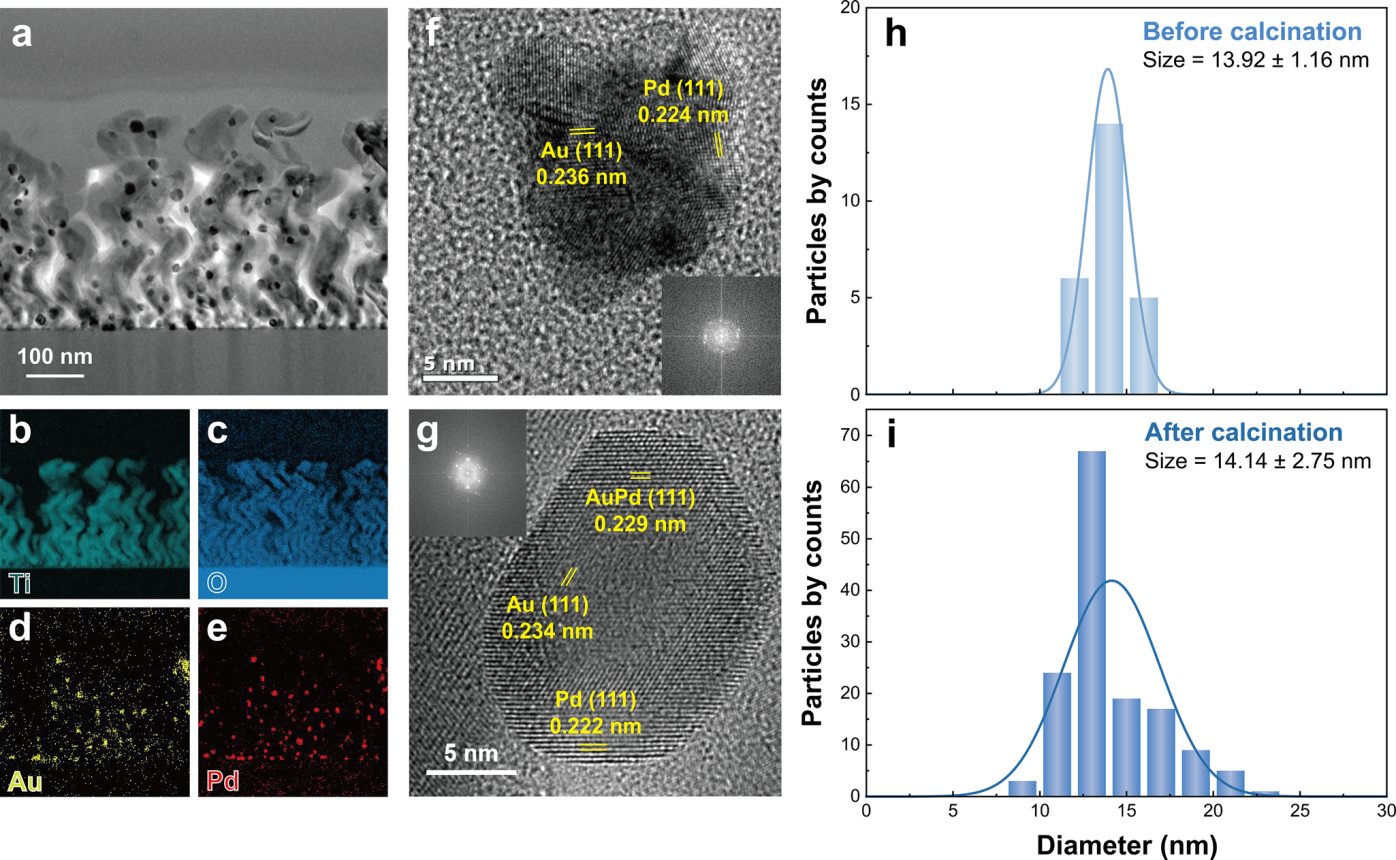
a) HR‐TEM images of Au@Pd NCs decorated TiO_2_ NHs at a 100 nm scale, along with the corresponding EDS elemental mapping images of b) Ti, c) O, d) Au, and e) Pd. HR‐TEM images of Au@Pd NCs on TiO_2_ NHs at a 5 nm scale, including d‐spacing information extracted from FFTs analysis: f) before and g) after calcination. Size distribution of Au@Pd NCs, with an average diameter of 13.92 nm h) before and 14.14 nm i) after calcination, obtained from HR‐TEM images.

**Figure** **3**a–e shows a high‐resolution transmission electron microscopy (HR‐TEM) image and corresponding energy‐dispersive X‐ray spectroscopy (EDS) images of an array of TiO_2_ NHs decorated with the Au@Pd NCs. NCs are uniformly dispersed throughout entire NHs and retain their composition without agglomeration or forming a monometallic phase, even after undergoing a harsh calcination process. (Figure S1, Supporting Information)

The effects of calcination were investigated by employing HR‐TEM imaging and size distribution analysis. Figure 3f shows HR‐TEM images of the Au@Pd NCs before calcination. The NCs exhibit a star‐shaped morphology, with Au and Pd in distinct phases. The measured d‐spacings are 0.236 nm for the (111) planes of Au and 0.224 nm for the (111) planes of Pd.^[^ ^29^, ^30^ ^]^ In Figure 3h, the size distribution of NCs, extracted from HR‐TEM images, follows a normal distribution, with an average size of ≈13.9 nm. On the other hand, Figure 3g depicts the HR‐TEM image of the Au@Pd NCs after calcination, confirming a d‐spacing of 0.229 nm for the (111) plane of the AuPd alloy, as well as for the (111) planes of both Au and Pd. Additionally, the inverse FFT images further demonstrate that distinct Pd shell covers the AuPd and Au core (Figure S2, Supporting Information). This unique core‐shell structure causes distinctive catalytic activity toward the toluene gas, which will be discussed in following sections. Moreover, the size distribution of NCs after calcination is depicted in Figure 3i, indicating an average size of 14.1 nm. It is noteworthy that the size of the NCs remains consistent before and after calcination, demonstrating that no agglomeration occurred, as also seen in Figures 3d and e, due to the well‐dispersion of the NCs on the TiO_2_ NHs using our method.

**Figure 4:**
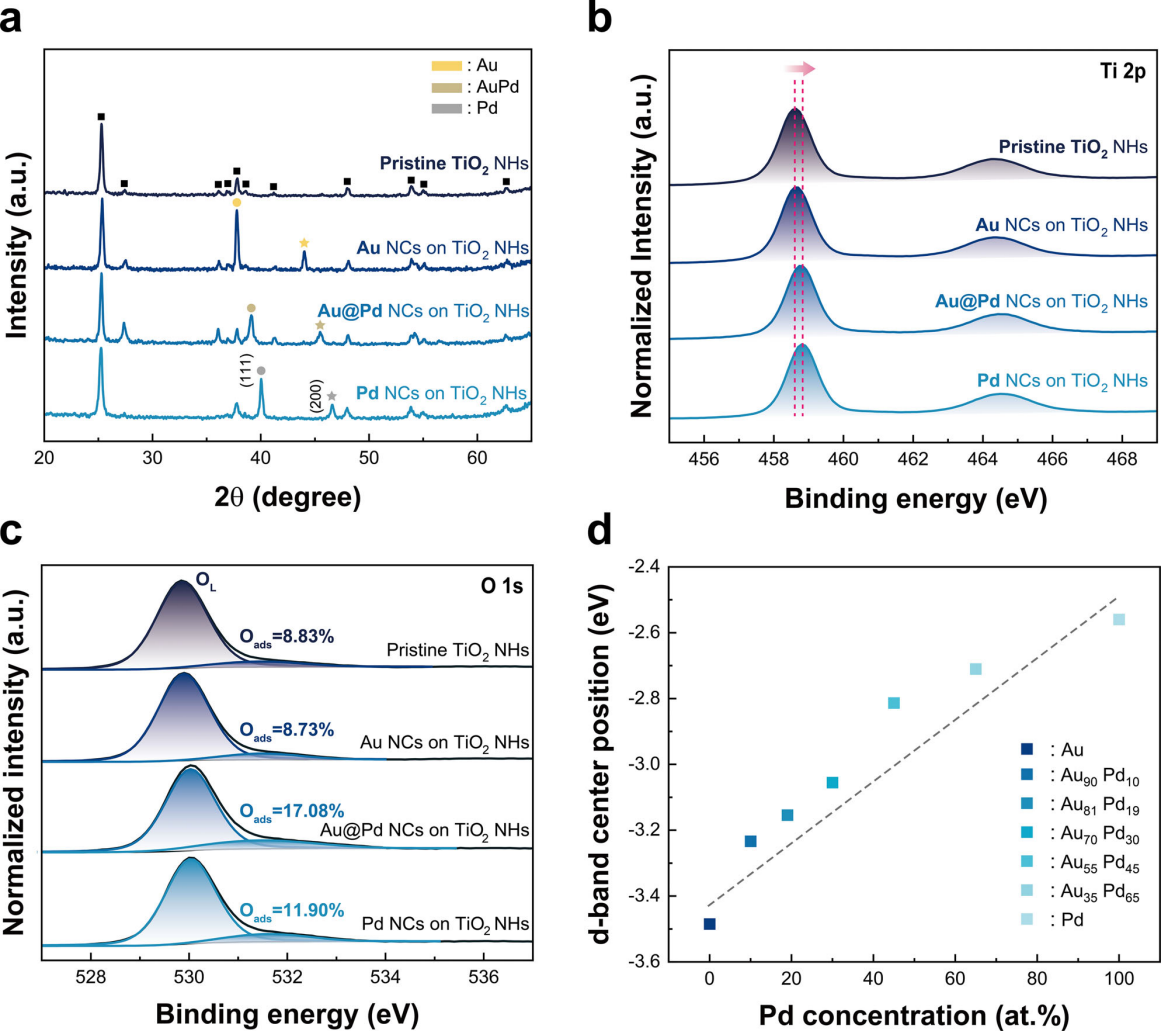
a) XRD spectrum of pristine TiO_2_ NHs, Au, Pd, and Au@Pd NCs decorated TiO_2_ NHs. XPS core level spectrum of b) Ti 2p, c) O 1s for the same sample set. d) The d‐band center positions of pristine TiO_2_ NHs, Au and Pd based NCs decorated TiO_2_ NHs.

The structural properties of the Au@Pd NCs on the TiO_2_ NHs array were evaluated using X‐ray diffraction (XRD) measurements. **Figure** **4**a shows the XRD spectra of pristine TiO_2_ NHs, Au NCs, Au@Pd NCs, and Pd NCs on TiO_2_ NHs. The pristine TiO_2_ NHs sample exhibits characteristic peaks of the anatase TiO_2_. For the Au NCs on TiO_2_ NHs sample, two characteristic peaks appear at 37.8° and 44.0°, corresponding to the (111) and (200) planes of Au, respectively. In the case of the Au@Pd NCs on TiO_2_ NHs sample, the shift to higher‐angle peaks at 39.1° and 45.4° suggests the formation of an AuPd alloy via Pd incorporation into the Au lattice.^[^ ^31^ ^]^ Furthermore, the Pd on TiO_2_ NHs samples shows additional peaks at 40.0° and 46.6°, corresponding to the (111) and (200) planes of Pd.

To investigate the chemical bonding properties, X‐ray photoelectron spectroscopy (XPS) was employed. Figure 4b shows the Ti 2p core‐level spectra at 458.6 and 464.3 eV which correspond to Ti 2p3/2 and Ti 2p1/2, respectively. The Ti 2p peak position is associated with Ti^4+^, and the absence of satellite peaks suggests that TiO_2_ has formed with a stoichiometric Ti:O ratio of 1:2. Compared with pure TiO_2_ NHs, the overall Ti 2p XPS spectra shifted toward high binding energy after the addition of NCs. In particular, the largest shift was observed by 0.2 eV after the addition of Pd NCs. It suggests that electrons migrate from TiO_2_ NHs to the NCs, inducing the formation of a depletion layer at the NCs/TiO_2_ interface. This led to the electronic sensitization of the TiO_2_ NHs, improving the sensitivity of the gas sensors. The O 1s spectra of four samples are shown in Figure 4c. The peak ≈530 eV is deconvoluted into two contributions, which can be assigned to lattice oxygen (O_L,_ ≈530.0 eV), and adsorbed oxygen (O_ads_, ≈531.4 eV). As oxygen is adsorbed on the surface of n‐type MOXs, electrons migrate from the MOXs surface to O_ads_, forming an electron depletion layer. When O_ads_ reacts with the target gases, captured electrons are released back to the MOXs surface, shrinking the depletion layer and increasing the conductivity of the MOXs. Therefore, in MOXs‐based gas sensors, O_ads_ is regarded as the reactive species responsible for the sensitivity of the gas sensor. In Figure 4c, the sample containing Pd exhibits a relatively high O_ads_ ratio, especially bimetallic Au@Pd NCs on TiO_2_ NHs exhibits the highest O_ads_ ratio of 17.08%. This result demonstrates that Pd plays an important role in facilitating adsorption of oxygen species onto the MOX surfaces, known as the oxygen spill‐over effect which can be further promoted by synergistic effect of bimetallic Au@Pd NCs.^[^ ^32^ ^]^ Chemical sensitization arising from the oxygen spill‐over suggests that Au@Pd NCs decorated TiO_2_ NHs have great potential as a highly sensitive gas sensing material.

The positions of the d‐band center were estimated for pristine TiO_2_, as well as for TiO_2_ decorated with monometallic and bimetallic NCs with various compositions, as depicted in Figure 4d. The d‐band center position is extracted by integrating the valence band spectra of each sample (Figure S3, Supporting Information). It has been reported that the d‐band center position is closely related to the adsorption energy of catalysts. As the d‐band center approaches the Fermi energy level (E_F_), the binding energy to the adsorbate strengthens; as it moves farther away from the E_F_, the binding energy weakens.^[^ ^33^, ^34^ ^]^ Compared to Au and TiO_2_, Pd possesses abundant anti‐bonding states, which implies that its d‐band center locates near the E_F_. Therefore, NCs with higher Pd composition show d‐band center positions closer to the E_F_. In fact, the d‐band center of the Pd on TiO_2_ sample is −2.56 eV below the E_F_, and as the Au composition increases, it moves further away, reaching −3.48 eV for the Au on TiO_2_ sample.

In general, a sensor with a stronger binding energy to the adsorbate exhibits a higher response. However, in our study, the Au_55_Pd_45_ NCs on TiO_2_ NHs showed significantly higher response to toluene gas than the Pd on TiO_2_ NHs, despite their deeper d‐band center position of −2.81 eV. These results imply that the d‐band center characteristics obtained from XPS valence spectrum do not fully represent the binding energy toward the adsorbate. We believe this inconsistency arises from the unique features of the core‐shell structure. To further investigate the correlation between the surface d‐band and the binding energy, we performed DFT calculations, which will be discussed in the final section.

### 2.3 Gas Sensing Performance

The performance of gas sensors was evaluated under dry air (N_2_ 79%, O_2_ 21%) conditions, with a 5 V voltage applied through the IDEs. The sensor response was calculated as the ratio between the baseline resistance (*R_a_*) and saturated resistance after gas exposure (*R_g_*), expressed as *R_a_*/*R_g_* *or* *R_g_*/*R_a_*. To determine the optimal operating temperature and the Au@Pd NCs composition, gas responses for 100 ppm toluene were tested at 150, 200, 250 °C with varying compositions of Au‐ and Pd‐based NCs, as illustrated in Figure S4 (Supporting Information). The Au_55_Pd_45_ composition exhibited consistently high sensitivity toward toluene, with optimal response at 200 °C. Below this temperature, limited surface activation hinders gas interaction, whereas above 200 °C, excessive desorption of chemisorbed oxygen diminishes surface reactivity and sensor performance.

**Figure 5:**
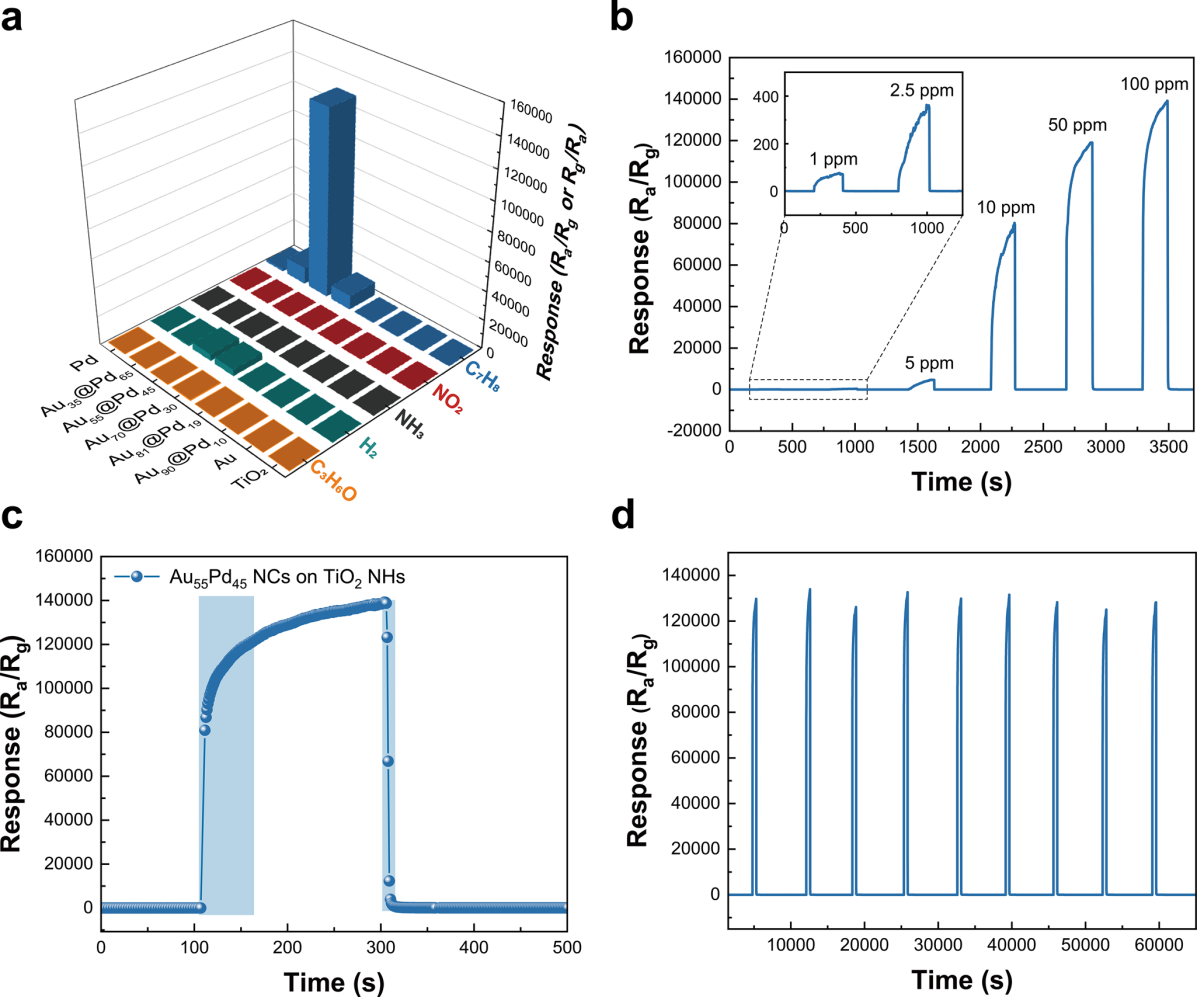
Gas sensing performance of Au@Pd NCs decorated TiO_2_ NHs, a) comparative gas responses of various samples to different gases, b) dynamic response curves for toluene concentrations ranging from to 1 to 100 ppm, c) response and recovery behavior upon exposure to 100 ppm toluene, and d) long–term stability test under repeated exposure to 100 ppm toluene.

To further assess sensor selectivity, both the Au@Pd NCs decorated and pristine TiO_2_ NHs sample were exposed to various gases – acetone (C_3_H_6_O), hydrogen (H_2_), ammonia (NH_3_), nitrogen dioxide (NO_2_), and toluene (C_7_H_8_) – under identical conditions (100 ppm, 200 s, 200 °C), as depicted in **Figure** **5**a. While the majority of the sensors decorated with Au@Pd NCs demonstrates sensitivity toward toluene gas, the Au_55_Pd_45_ sample stands out for its outstanding selectivity toward toluene compared to the other sensors. Figure S5 (Supporting Information) provides a log‐scale bar chart comparing the responses of the Au_55_Pd_45_ sample and pristine TiO_2_ NHs. The pristine TiO_2_ NHs gas sensor generally shows low response to all test gases. Its selectivity (*S*_*Tol*/*Ace*_) toward toluene is low as well, with a ratio of 2.7 relative to acetone. Notably, the response of the Au_55_Pd_45_ sample reaches 131013 for 100 ppm toluene. The toluene selectivity over acetone (*S*_*Tol*/*Ace*_) reaches 1008, exhibiting 373‐fold increase compared to pristine TiO_2_ NHs sample. Achieving high selectivity over acetone is essential for real‐world applications, yet remains challenging due to the chemical and structural similarity between acetone and toluene.^[^ ^35^ ^]^ Nevertheless, the Au_55_Pd_45_ sample exhibited remarkably high selectivity, surpassing the previously reported value by a large margin.^[^ ^36^, ^37^, ^38^, ^39^, ^40^, ^41^, ^42^, ^43^ ^]^ This remarkable enhancement in response to toluene gas results from the electronic and chemical sensitization effects of the bimetallic Au@Pd NCs, as previously demonstrated by XPS analysis. Furthermore, the high selectivity is attributed to the outstanding homogeneity of the bimetallic Au@Pd NCs incorporated into the TiO_2_ NHs.

Figure 5b shows dynamic response of toluene gas concentrations ranging from 1 to 100 ppm. Using the corresponding response values on the y‐axis, the calibration curve is presented as a log‐log plot, with concentration on the x‐axis (Figure S6, Supporting Information). The calibration curve for toluene gas ranging from 1 to 100 ppm can be extrapolated using the linear relationship *y*  =  3.06*x* + 1.64. Based on the linear regression curves and standard deviation of the 30 blank signals (σ  =  0.004855, as shown in Table S1, Supporting Information), we can estimate the limit of detection (LoD) value to be 73.1 ppb, which is much lower than the guideline value for indoor toluene concentration.

For the practical applications, it is essential to develop gas sensors that offer reliable operation, with fast response‐recovery times and stability under repeated exposure to gases. Response time, measured during exposure to 100 ppm toluene at 200 °C, is defined as the time required to reach 90% of the saturation current from the baseline current, while the recovery time is defined as the time required to return to baseline current after the exposure. The response‐recovery times were recorded as 69 and 4 s, respectively, both of which are regarded as remarkably fast at the relatively low operating temperature of 200 °C (Figure 5c). This rapid response‐recovery property is associated with abundant ionized oxygen species on the TiO_2_ NHs surfaces, which accelerate the avalanche reaction upon introduction of toluene molecules.^[^ ^44^ ^]^ In fact, as confirmed by the O 1s spectra, the decoration of Au@Pd NCs led to a notable increase in O_ads_ species, supporting its strong correlation with the rapid response and recovery times. Stability of the Au@Pd decorated TiO_2_ gas sensors also measured under the same toluene gas exposure conditions. As illustrated in Figure 5d, the sensor exhibits stable operation under 9 repeated toluene gas exposures, with a total test duration of 60 000 s.

### 2.4 Unraveling the Underlying Mechanism of Highly‐Selective Toluene Detection

**Figure 6:**
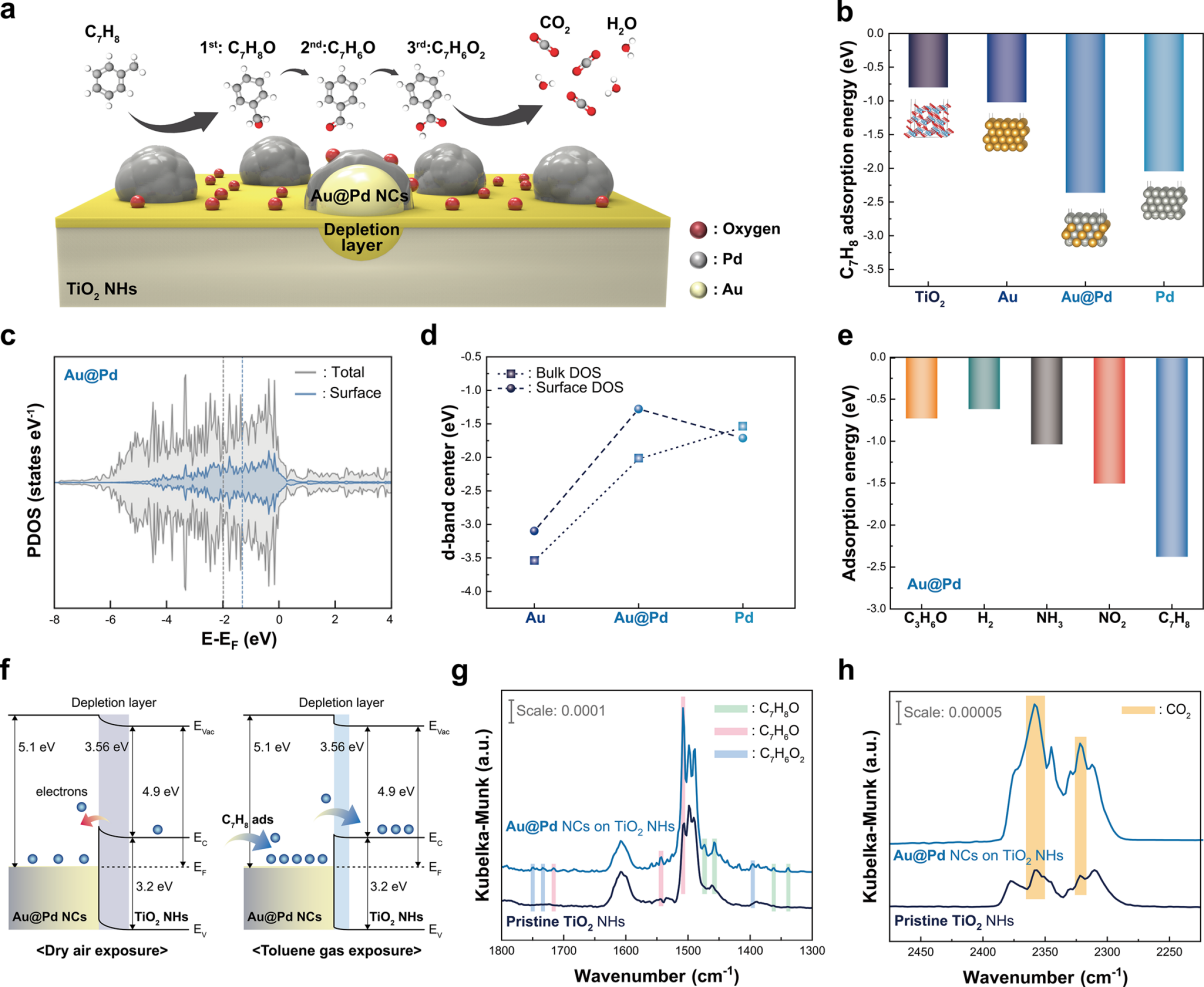
a) Schematic illustration of overall reaction process of toluene gas on the Au@Pd NCs decorated TiO_2_ surface. b) Comparative toluene adsorption energies on TiO_2_, Au, Au@Pd and Pd. c) PDOS of the entire slab model and surface atomic layer of Au@Pd NCs. d) Comparison of d‐band center positions between bulk and surface atoms for Au, Pd, and Au@Pd NCs. e) Calculated adsorption energies of five gases on Au@Pd surface. f) Schematic illustration of band diagram before and after toluene adsorption. In situ DRIFTS spectra of g) toluene oxidation intermediates‐related region (1300–1800 cm^−1^) and h) CO_2_‐related region (2225–2475 cm^−1^) for Au@Pd NCs on TiO_2_ NHs and pristine TiO_2_ NHs.

Density functional theory (DFT) calculations were employed to investigate the origin of the abnormally high toluene selectivity exhibited by Au@Pd NCs decorated on TiO_2_ NHs. As the gas sensor response primarily governed by the adsorption energy of gas molecules on sensing materials, a comparative analysis was performed across different NC compositions to identify the origin of the enhanced adsorption characteristics of the bimetallic Au@Pd NCs.^[^ ^45^, ^46^, ^47^, ^48^ ^]^ Subsequently, to elucidate the selective response toward toluene, the adsorption behaviors of five different gas molecules were examined. Furthermore, to gain deeper insights into the toluene sensing mechanism, in situ diffuse reflectance infrared Fourier transform spectroscopy (DRIFTS) was employed to identify the intermediates and by‐products formed on the sensor surface. For conceptual understanding, the overall reaction process is schematically illustrated in **Figure** **6**a.

First, the adsorption energy of toluene was calculated on TiO_2_, monometallic Au, Pd, and bimetallic Au@Pd surfaces to compare their relative affinities. Anatase TiO_2_ (101) surface was chosen based on XRD results showing a dominant (101) orientation, and FCC (111) surfaces for Au, Pd, and Au@Pd NCs were constructed, as XRD and HR‐TEM revealed (111) facets as the most exposed surface planes. For the Au@Pd NCs, which consists of a Pd shell overlying an AuPd alloy core, a slab model was developed with a Pd surface layer and subsurface AuPd layers, as illustrated in Figure S7 (Supporting Information).^[^ ^49^ ^]^

Figure 6b presents the calculated adsorption energies for toluene on the TiO_2_, Au, Au@Pd and Pd surfaces. Toluene exhibited the highest adsorption energy of −2.36 eV on the Au@Pd NC surface. This strong interaction facilitates the oxidation reaction of toluene on the surface, releasing a large number of electrons into TiO_2_ and resulting in a significantly enhanced sensor response.

To investigate the origin of the stronger adsorption on Au@Pd, we analyzed the projected density of states (PDOS) of surface, particularly focusing on the d‐orbital distribution of surface atoms. As mentioned earlier, d‐band position determines the strength of the bonding energy. Au exhibited d‐bands positioned deeply relative to the E_F_, resulting in weak adsorption, while Pd had shallow d‐bands, leading to stronger adsorption. Interestingly, the surface Pd layer in Au@Pd exhibited d‐bands shallower than those of Pd, enabling even stronger adsorption. While the total PDOS for Au@Pd spans a wide energy range due to contributions from both Au and Pd, the d‐band of the surface Pd layer is relatively concentrated in a higher energy region, as shown in Figure 6c. Figure 6d illustrates the d‐band centers for bulk and surface layers of Au, Pd, and Au@Pd NCs. (See Figure S8, Supporting Information for the corresponding PDOS profiles.) The d‐band center of the bulk AuPd alloy lies between those of pure Au and Pd. However, the d‐band center of the surface layer in Au@Pd is shifted closer to the E_F_ than that of pure Pd. This deviation can be attributed to the tensile strain induced in the surface Pd layer by the underlying AuPd alloy layers, which have larger lattice parameters. The tensile strain compresses the d‐band width of the surface Pd layer, shifting its d‐band center toward higher energy.^[^ ^50^ ^]^ (Table S2, Supporting Information)

To further assess the selective adsorption behavior of the Au@Pd‐decorated TiO_2_ NHs system toward toluene, adsorption energies of the five different gas molecules were calculated. Figure 6e shows the adsorption energies for the five gas molecules on the Au@Pd surfaces. (See Figure S9, Supporting Information for a full comparison across all four surfaces–TiO_2_, monometallic Au, Pd, and bimetallic Au@Pd. The optimized adsorption geometries are provided in Figures S10 and S11, Supporting Information)

Among the five gases, toluene exhibited the highest adsorption energy of −2.36 eV, confirming its strong and selective interaction with the Au@Pd surface. This strong adsorption facilitates efficient electron transfer from toluene, a reducing gas, to the TiO_2_ surface, leading to an increase in charge carriers and an enhanced sensor response (Figure 6f). Although NO_2_ showed the second‐highest adsorption energy, its sensor response was the lowest among the test gases. This discrepancy arises from its role as an oxidizing behavior, which withdraws electrons from the sensor surface, as demonstrated in Figure S12 (Supporting Information). It is previously reported that the sensing response to oxidizing gases such as NO_2_ is not improved since they do not enhance the electric conduction because of electron‐withdrawing characteristics.^[^ ^51^, ^52^ ^]^ In conclusion, the strong adsorption of toluene enhances sensing response by donating electrons to the surface, and thus toluene exhibits high selectivity on the Au@Pd NCs decorated TiO_2_ NHs.

Next, complementing the calculated adsorption behavior of toluene, in situ DRIFTS spectroscopy was employed to experimentally validate the overall sensing mechanism. Figure 6g compares the DRIFTS results of the Au@Pd NCs decorated TiO_2_ NHs and pristine TiO_2_ NHs after 30 min of continuous toluene gas exposure at 200 °C. The spectra in the range of 1300–1800 cm^−1^ exhibit multiple intermediate peaks associated with oxidized toluene species. Accordingly, a comparison of the two spectra allows for evaluating the differences in intermediate formation between the two samples upon exposure to toluene. Successive oxidation of toluene is evidenced by a series of distinct vibrational bands. Benzyl alcohol (C_7_H_8_O), the primary oxidation product, is identified by bands at 1473 and 1455 cm^−1^, along with υ(O–H) stretching at 1362 and 1338 cm^−1^. Subsequent formation of benzaldehyde (C_7_H_6_O) is confirmed by υ(C═O) bands at 1506, 1541, and 1717 cm^−1^. Further oxidation to benzoic acid (C_7_H_6_O_2_) is supported by the presence of peaks at 1748, 1732, and 1396 cm^−1^.^[^ ^53^ ^]^ These intermediate peaks are more prominent in the spectrum of the Au@Pd NCs decorated TiO_2_ NHs than in pristine TiO_2_, suggesting that Au@Pd NCs significantly facilitate the rapid oxidation of toluene. Moreover, to investigate carbon dioxide (CO_2_) vibrational modes corresponding to the complete oxidation of toluene, spectra in the 2225–2475 cm^−1^ region were collected after a 10‐min mild purge (10 sccm) for each sample, as illustrated in Figure 6h. Distinct asymmetric peaks at 2321 and 2358 cm^−1^ correspond to CO_2_ vibrational modes, confirming the complete oxidation of toluene.^[^ ^54^ ^]^ This oxidation process enables increased electron transfer from toluene to the sensor surface, leading to an enhanced response. Notably, the higher CO_2_ peak intensity observed in the Au@Pd NCs decorated TiO_2_ NHs suggests more efficient oxidation, consistent with DFT calculation results for strong adsorption of toluene on the Au@Pd NCs.

## 3 Conclusion

We demonstrated highly toluene‐selective MOX‐based gas sensors decorated with homogeneous Au‐Pd bimetallic NCs (Au@Pd) synthesized via seed‐mediated sequential reduction of Au and Pd on an array of TiO_2_ NHs matrix. TEM characterization revealed a high degree of homogeneity and uniform spatial distribution of the NCs. Owing to the meticulous fabrication process, the optimized gas sensor (Au_55_Pd_45_ NCs decorated on TiO_2_ NHs) exhibited an exceptionally high response of ≈130 000 to 100 ppm toluene, a low LoD of 73.1 ppb, and fast response and recovery times of 69 s and 4 s, respectively. The excellent sensitivity toward toluene is attributed to the chemical and electrical catalytic effects induced by the decoration of bimetallic Au@Pd NCs on the surface of TiO_2_ NHs. Chemical sensitization, which promotes the oxygen spill‐over effect onto the surface of the TiO_2_ NHs, is verified by O 1s core‐level spectra. Concurrently, electrical sensitization, manifested as a broadened depletion region at the NCs/TiO_2_ NHs interface, is confirmed through Ti 2p core‐level analysis. Additionally, DFT calculations revealed that the surface electronic structure of the Au@Pd NCs is influenced by their unique core–shell configuration, resulting in a d‐band center shift closer to the E_F_, which enhances gas adsorption strength. This active NCs surface exhibited a preferential affinity for toluene over other interfering gases. Furthermore, in situ DRIFTS offered direct evidence for the presence of toluene oxidative intermediates and demonstrated that incorporated Au@Pd NCs promote complete toluene oxidation, thereby validating the theoretical predictions and the DFT calculation results. The homogeneous and uniform nature of the NCs also contributes to the formation of consistent and well‐defined active sites. As a result, a gas sensor decorated with homogeneous, core‐shell‐structured Au@Pd NCs demonstrates exceptionally high selectivity for toluene, with a 1008‐fold higher response compared to that for acetone.

## 4 Experimental Section

### Au, Au@Pd and Pd NCs Synthesis

To synthesize bimetallic Au@Pd nanocatalysts (NCs), gold nanocatalysts (Au NCs) should be synthesized first to make the core of the bimetallic NCs. The synthesis process of Au NCs was described briefly as follows. First, 5 mL of 50 mM trisodium citrate (Na_3_Ct) as a reducing agent was stirred with deionized water (DI water, 94 mL), which was heated to boil with vigorous stirring. Then, 1 mL of Gold (III) chloride hydrate (HAuCl_4_, 15 mg mL^−1^) was added to the boiling Na_3_Ct solution. The mixture was stirred for 8 min until the color of the solution changed from light purple to wine red. The final Au NCs solution (0.44 mM) was filtered using a 0.22 µm poly(vinylidene fluoride) (PVDF) syringe filter to remove some aggregated particles. The synthesized Au NCs solution was stored at 4 °C until further use.^[^ ^55^ ^]^ To grow Pd nanocluster on the surface of the Au NCs, 1.115 mL of 4 mM Sodium tetrachloropalladate(II) (Na_2_PdCl_4_) and 10.765 mL of 0.1 M L‐ascorbic acid were added to 15 mL of the Au NCs seed solution.^[^ ^56^ ^]^ The mixture was stirred at room temperature for 15 min. For preparing different composition ratios of Au:Pd, all reaction parameters were unchanged except for the concentration of Na_2_PdCl_4_.

The resulting solution was then filtered using a 0.22 µm PVDF syringe filter. Subsequently, 1.2 mL of methoxy polyethylene glycol‐thiol (mPEG‐SH, molecular weight = 10KDa, 2.14 mg mL^−1^) was added drop by drop to the synthesized Au@Pd solution and stirred for 18 h 30 min to achieve surface functionalization.^[^ ^57^ ^]^ The mPEG‐SH conjugated on the surface of the Au@Pd NCs serves to prevent nanoparticle aggregation, particularly during high‐speed centrifugation step.

Pd NCs were synthesized for the control group test. First, 3 mL of 4.4 mM Sodium tetrachloropalladate(II) (Na_2_PdCl_4_), 45 mg of 44 mM Sodium Bromide (NaBr), and 0.15 mL of Cetyltrimethylammonium chloride (CTAC) were mixed in deionized water. The final volume of solution was adjusted to 10 mL.^[^ ^58^ ^]^ In this method, NaBr acted as a shape‐controlling agent and CTAC was served as a surfactant and a mild reducing agent.^[^ ^59^ ^]^ The mixture was then incubated in an oil bath at 90 °C for 48 h. During this period, the color of the solution changed from orange to dark brown. After completing this process, the final Pd NCs solution was filtered and centrifuged to remove remaining CTAC, and the concentration was diluted until it matched that of the Au NCs synthesized previously.

### TiO_2_ Nanohelices Deposition

The SiO_2_ substrate was sonicated sequentially in acetone, isopropyl alcohol (IPA), and double‐distilled water for 3 min to obtain a clean surface. Next, interdigitated electrodes (IDEs) were patterned by photolithography and Ti/Pt (5 nm/20 nm) were deposited using an electron beam evaporator at a deposition rate of 0.5 Å s^−1^, forming an active area of 200 × 200 µm^2^. Subsequently, TiO_2_ NHs were directly deposited on the IDEs using the OAD method.^[^ ^60^ ^]^ During the OAD process, the deposition rate was maintained at 1.5 Å s^−1^ under a constant base pressure of 3.0 × 10^−6^ Torr.^[^ ^61^ ^]^ The vapor flux incidence angle with respect to the substrate normal was adjusted at 80° to ensure the porosity of TiO_2_ NHs.^[^ ^62^ ^]^ To form a helical structure, the substrate was rotated every 2 s at 1 rpm and then stopped for 12 s.^[^ ^63^ ^]^

### NCs Decoration on TiO_2_ NHs Matrix

As‐prepared bimetallic mPEG‐Au@Pd NCs were collected by centrifugation at 14 000 rpm for 30 min and re‐dispersed in DI water for storage at 4 °C. Prior to use, the stored product was centrifuged again and re‐dispersed in 30% (v/v) ethanol.^[^ ^64^ ^]^ A final centrifugation was then performed to concentrate the suspension. Approximately 40 µL of the concentrated NC solution was drop‐cast onto TiO_2_ NHs, followed by drying in an oven at 100 °C for 2 h to remove the solvent. In the final step, rapid calcination was performed at 700 °C for 30 s under a the reducing N_2_ atmosphere to thermally decompose mPEG and securely immobilize the Au@Pd NCs onto TiO_2_ NHs. For comparison, pristine TiO_2_ NHs also calcined under the same conditions without drop‐casting the NC solution.

### Structural Characterization

The morphology and elemental distribution of Au@Pd NCs decorated TiO_2_ NHs were examined using cross‐sectional high resolution scanning transmission electron microscopy (HR‐STEM, ARM‐200F, JEOL Ltd., Japan) operated at 80 kV with a probe corrector (ASCOR, CEOS GmbH, Germany) at the Materials Imaging & Analysis Center of POSTECH. To characterize the crystalline structure of the NCs on the TiO_2_ NHs, XRD spectra were collected using an X‐ray diffractometer (Rigaku, D/MAX‐2500) with Cu K𝛼 radiation (𝜆 = 1.5418 Å). The surface chemical states of the catalysts and TiO_2_ matrix were were analyzed by XPS (Thermo FisherScientific Co.) using a monochromated Al K𝛼 radiation source.

### Gas Sensing Measurement

Prior to measurement, the gas sensors were aged at 200 °C for 2 hou under a constant flow of 500 sccm dry air (N_2_ 79%, O_2_ 21%). During the measurement, 5 V was applied across the IDEs using a Keithley 4200 Source Measurement Unit (SMU). Parallel gas lines for dry air and mixed gases (each at 500 sccm) were connected to the chamber through a 4‐way valve operated by a toggle switch. The mixture gas exposure was maintained for 200 s to allow the sensor current to reach saturation, and the refreshing process continued until the current returned to its baseline.

### Computational Details

Density functional theory (DFT) calculations were performed using the Vienna Ab initio Simulation Package (VASP).^[^ ^65^ ^]^ The generalized gradient approximation (GGA) with the Perdew‐Burke‐Ernzerhof (PBE) functional was applied for exchange‐correlation interactions, and a plane wave cutoff energy of 520 eV was employed.^[^ ^66^ ^]^ Structural optimizations were performed until the forces on atoms were less than 0.01 eV Å^−1^, and the total energy was converged to within 10^−6^ eV during self‐consistent field (SCF) iterations. Van der Waals interactions were included using the Grimme‐D3 dispersion correction method.^[^ ^67^ ^]^ For anatase TiO_2_ (101), the surface model comprised three atomic layers, where the bottom layer was fixed, and the top two layers were allowed to relax. To eliminate interactions between periodically repeated slabs, a (3 × 1) supercell with a vacuum region of ≈15 Å along the z‐axis was constructed. The k‐point sampling was performed using a Gamma‐centered Monkhorst‐Pack grid with a 3 × 3 × 1 resolution, adjusted to accommodate the supercell's lattice parameters. For Au, Pd, and Au@Pd (111) surfaces, four‐layer slab models were used, with the bottom two layers fixed and the top two layers relaxed. A (3 × 3) supercell was constructed, and a 4 × 4 × 1 Gamma‐centered k‐point grid was applied. The gas adsorption energy was calculated as the difference between the total energy of the surface with the adsorbed gas molecule and the sum of the energies of the clean surface and the isolated gas molecule.

(1) $$\;E_{\mathrm{adso}\mathrm{rpti}\mathrm{on}}=E_{\mathrm{surf}\mathrm{ace}+\mathrm{adsorbed}\mathrm{gas}\mathrm{molecule}}\;-E_{\mathrm{surf}\mathrm{ace}}-E_{\mathrm{gas}\;\mathrm{mole}\mathrm{cule}}$$

In situ *diffuse reflectance infrared Fourier transform spectroscopy (DRIFTS)*: In situ DRIFTS experiments were performed using a Nicolet iS‐50 FTIR spectrometer (Thermo Fisher Scientific) equipped with a mercury cadmium telluride (MCT) detector, a diffuse reflectance cell, and a high temperature reaction chamber (HTV) with KBr windows. The spectra were recorded from 400 to 4000 cm^−1^ with 64 scans and a spectral resolution of 4 cm^−1^. Before each measurement, samples were placed in the diffuse reflectance cell and high‐purity air (99.999%) was continuously purged into the cell at 250 °C (523 K) for 1 h. Temperature was maintained at 200 °C (473 K) throughout the experiment and the background spectra were obtained after purging. Toluene was introduced into the cell by bubbling air for 30 min and then air was injected into the cell for purging.

## Conflict of Interest

The authors declare no conflict of interest.

## Supporting information



Supporting Information

## Data Availability

The data that support the findings of this study are available from the corresponding author upon reasonable request.
