# Supplementary material for: TiO2 Nanohelices Decorated with Homogeneous Au‐Core Pd‐Shell Nanocatalysts for Selective Toluene Gas Detection
Source: Small. 2025 Jul 6;21(34):2504976. doi: 10.1002/smll.202504976 (PMC12393013; doi:10.1002/smll.202504976)
Supplement: Supplementary file 1 — Supporting Information [file SMLL-21-2504976-s001.docx]

Supporting Information

TiO_2_ Nanohelices Decorated with Homogeneous Au-Core Pd-Shell Nanocatalysts for Selective Toluene Gas Detection

Hyeonwoong Hwang, Hanseo Bae, Eunji Ahn, Dongmin Lee, Hyeon Ho Cho, Sunah Cheong, Tae Yeon Kim, Jaerim Kim, Yongju Yun, Donghwa Lee, Sei Kwang Hahn* and Jong Kyu Kim*


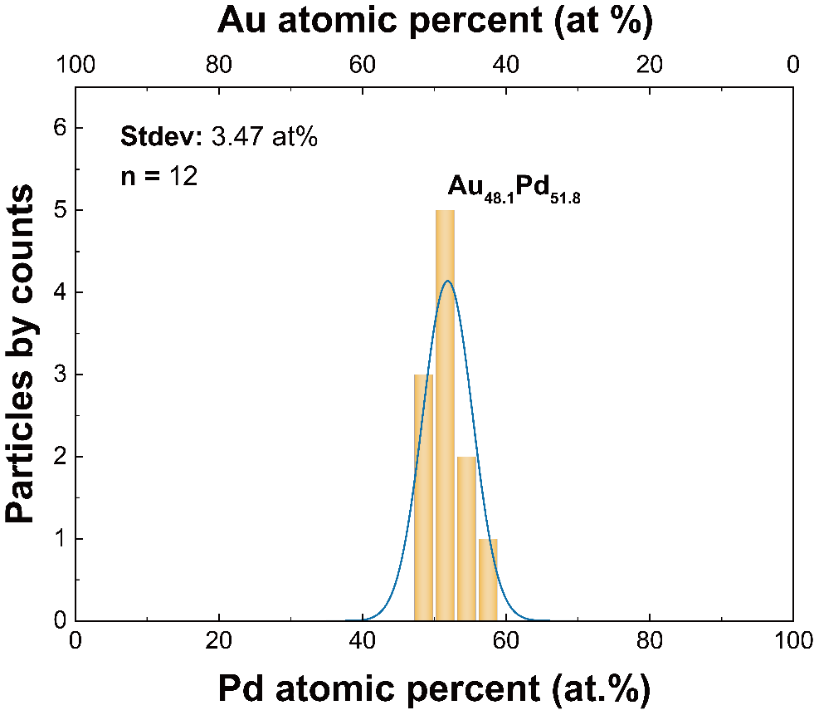


**Figure S1**. Atomic composition of Au_55_Pd_45_ NCs decorated on TiO_2_ NHs. Composition information extracted from STEM EDS elemental mapping images (n = 12)


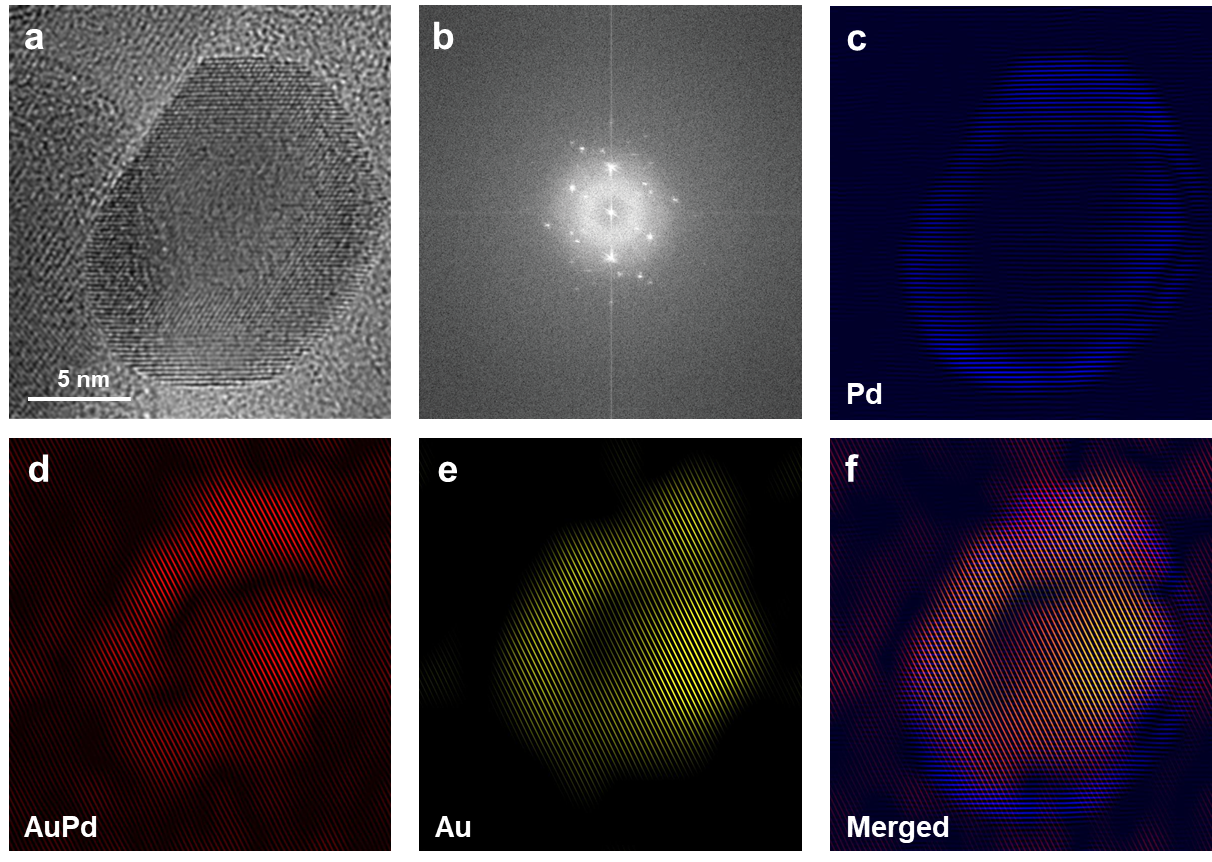


**Figure S2**. (a) TEM image of Au@Pd NCs decorated on TiO_2_ NHs on 5 nm scale, (b) corresponding FFT images, and inverse FFT images selected by (c) (111) spots of Pd, (d) (111) spots of AuPd, (e) (111) spots of Au and (f) merging image of (c), (d) and (e).


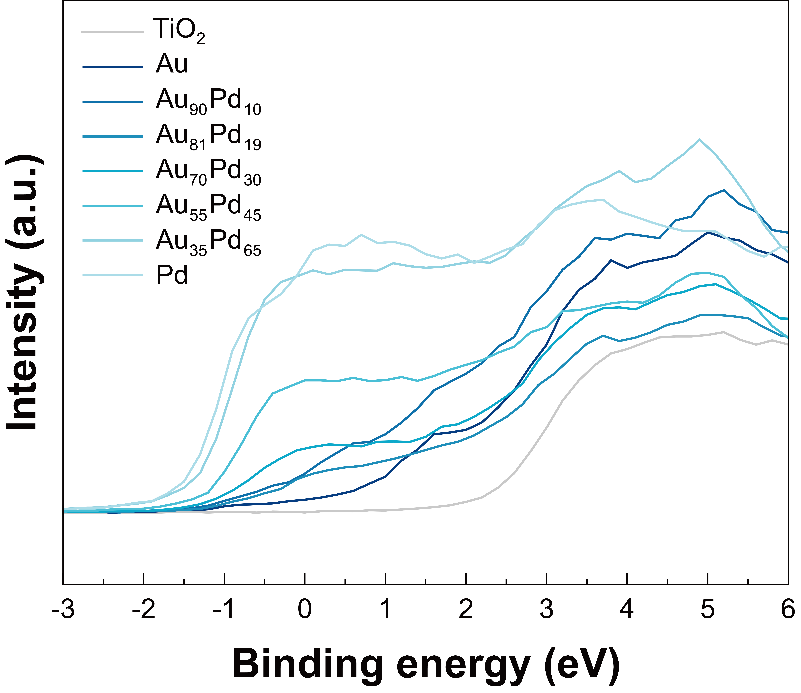


**Figure S3**. Valence band spectra of pristine TiO_2_ NHs, TiO_2_ NHs decorated with monometallic (Au, Pd) NCs, and TiO_2_ NHs decorated with bimetallic NCs of various compositions.


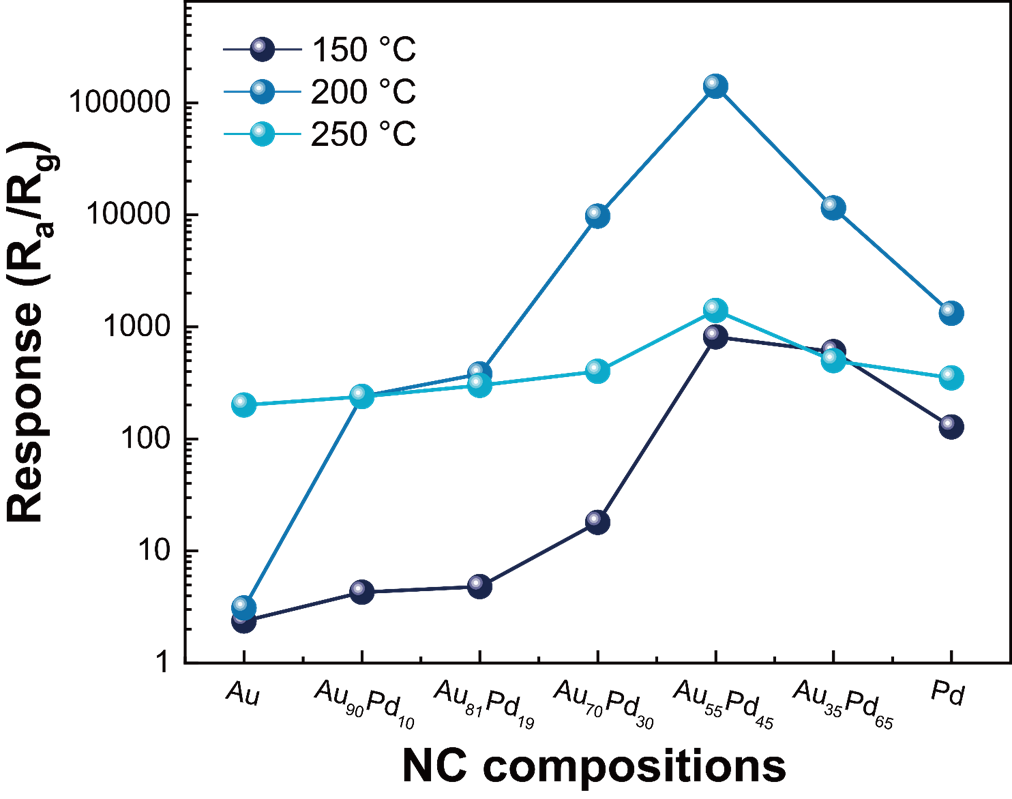


**Figure S4**. Log-scale plot for toluene (100 ppm) gas response measured at different operating temperatures (150 °C, 200 °C, 250 °C) for various compositions of Au-and Pd-based NCs decorated TiO_2_ NHs gas sensors.


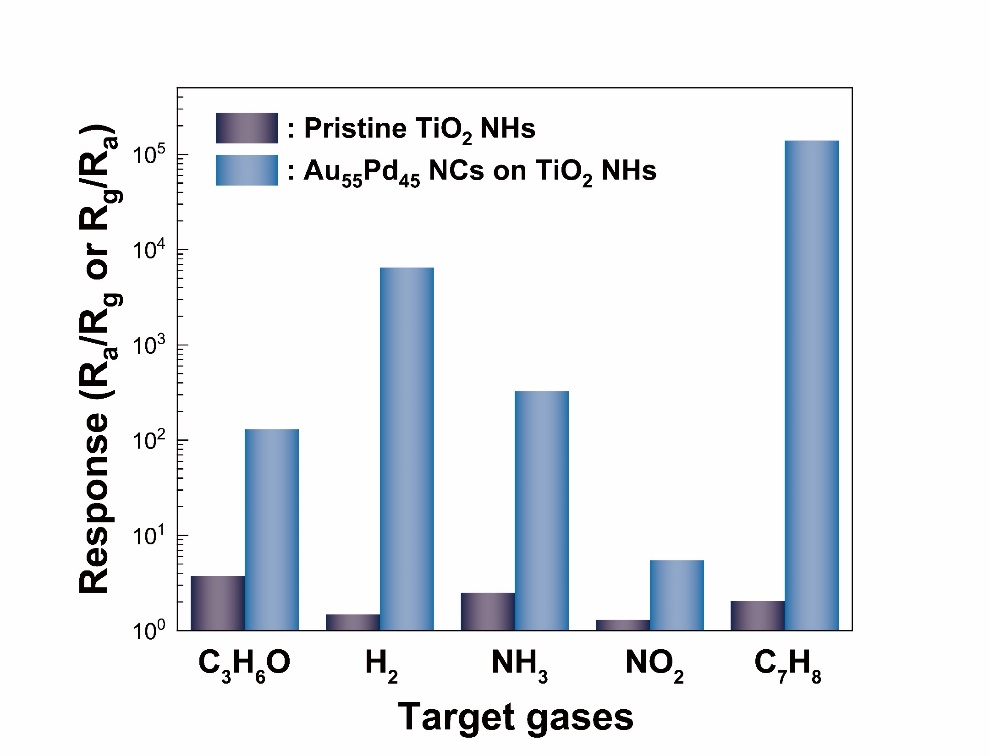


**Figure S5**. Log-scale bar chart of comparing gas response of the optimized Au_55_Pd_45_ NCs decorated TiO_2_ NHs with pristine TiO_2_ NHs. (Gas exposure conditions: 100 ppm, 200 C, 200 s)


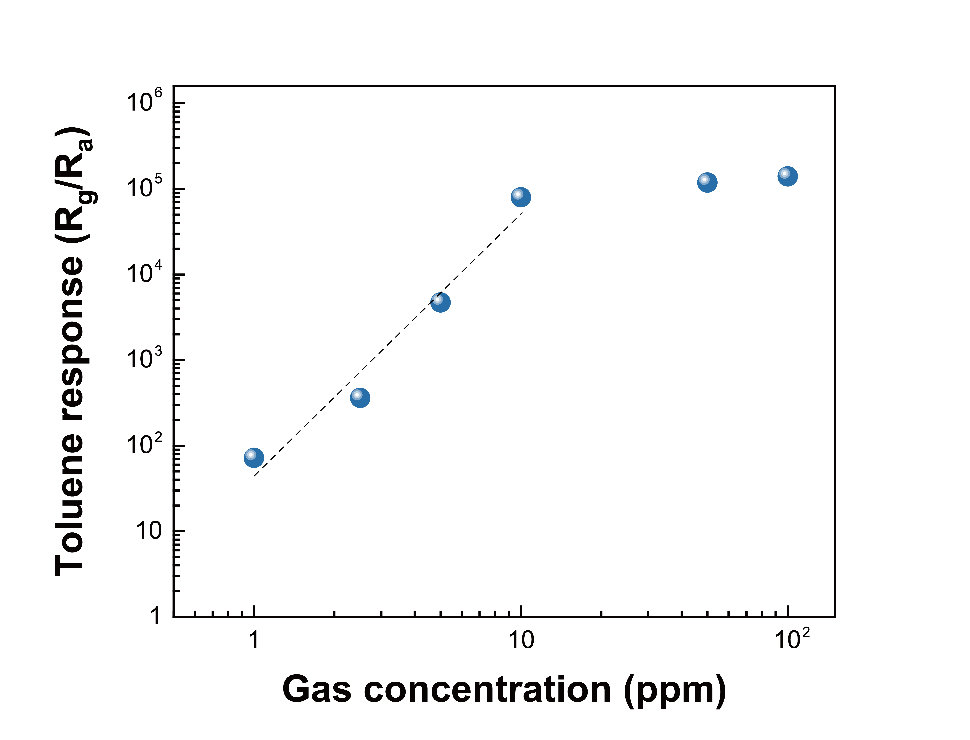


**Figure S6**. Log-scale calibration curve of the optimized Au_55_Pd_45_ NCs decorated TiO_2_ NHs gas sensor for toluene gas.


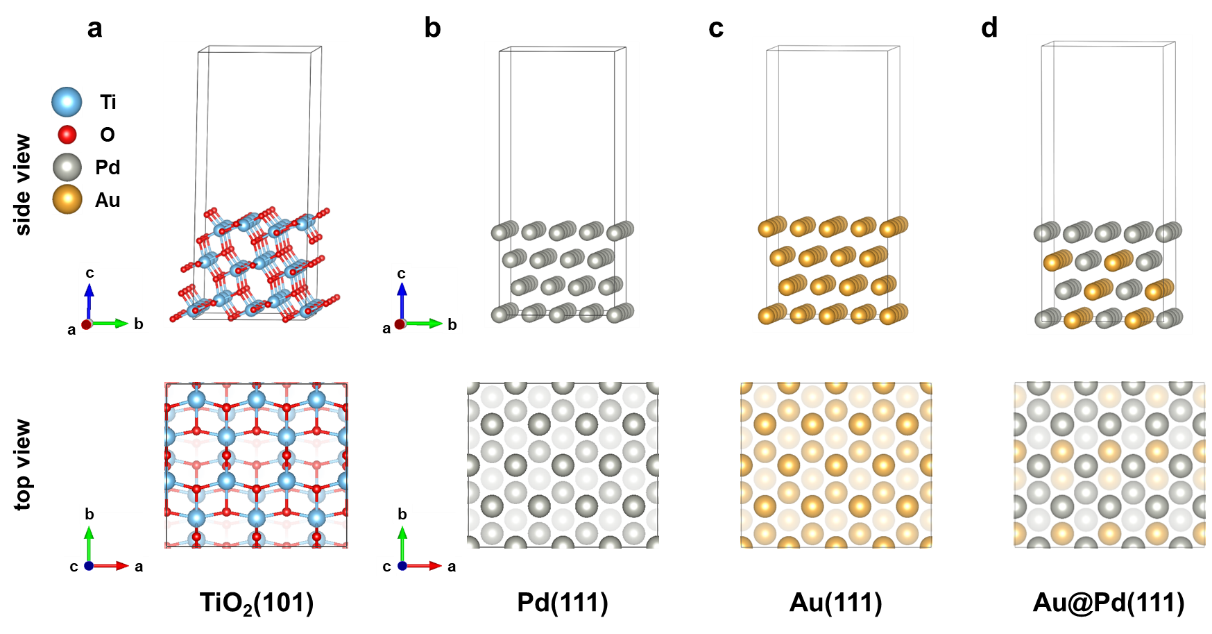


**Figure S7.** (a) Side and top view of anatase TiO_2_ (101), (b) Pd (111), (c) Au (111) and (d) Au@Pd (111) surface models.

**
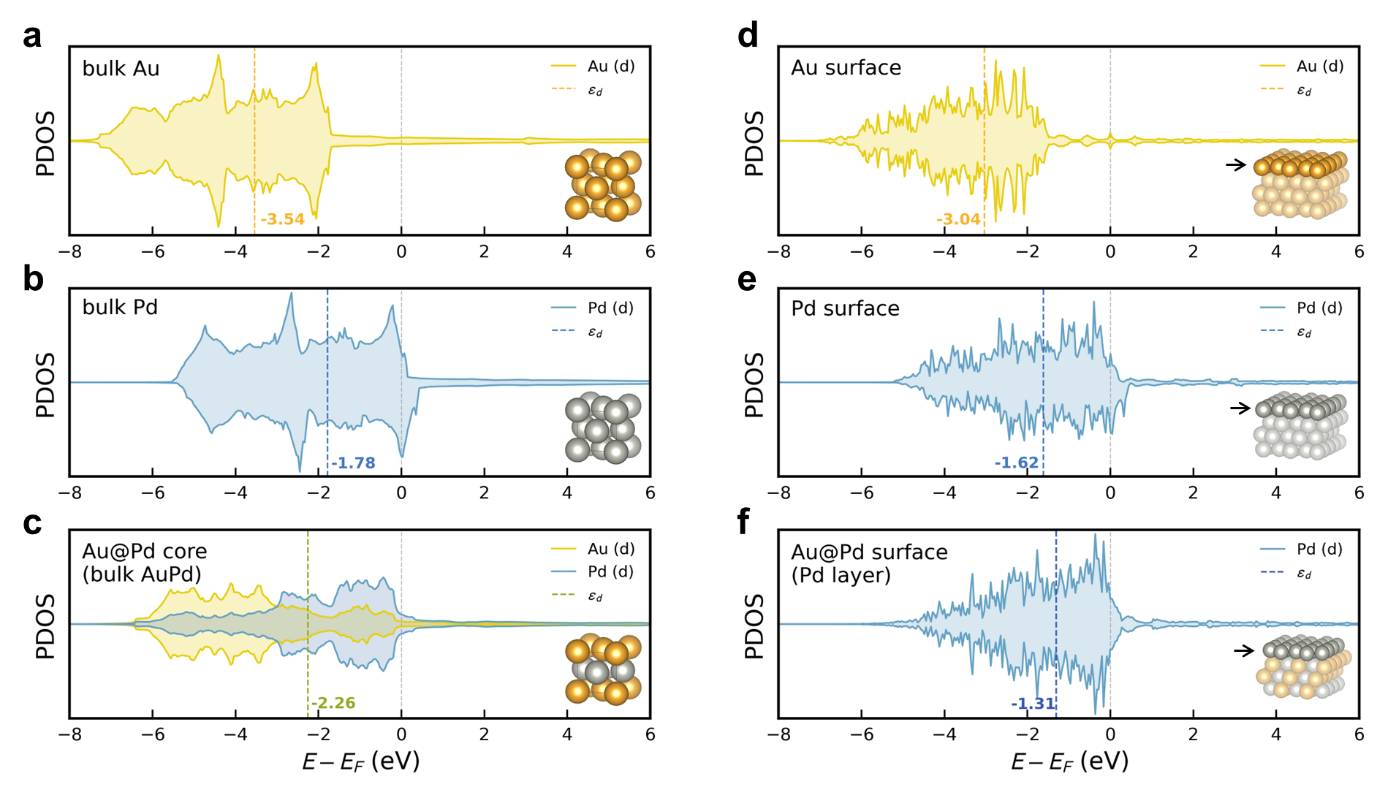
**

**Figure S8.** (a) Projected density of d states (PDOS) of bulk Au, (b) Pd and (c) AuPd alloy; (d) PDOS of surface layer for Au, (e) Pd and (f) Au@Pd NCs surfaces. d-band centers (ε_d_) are marked as coloured dotted lines.


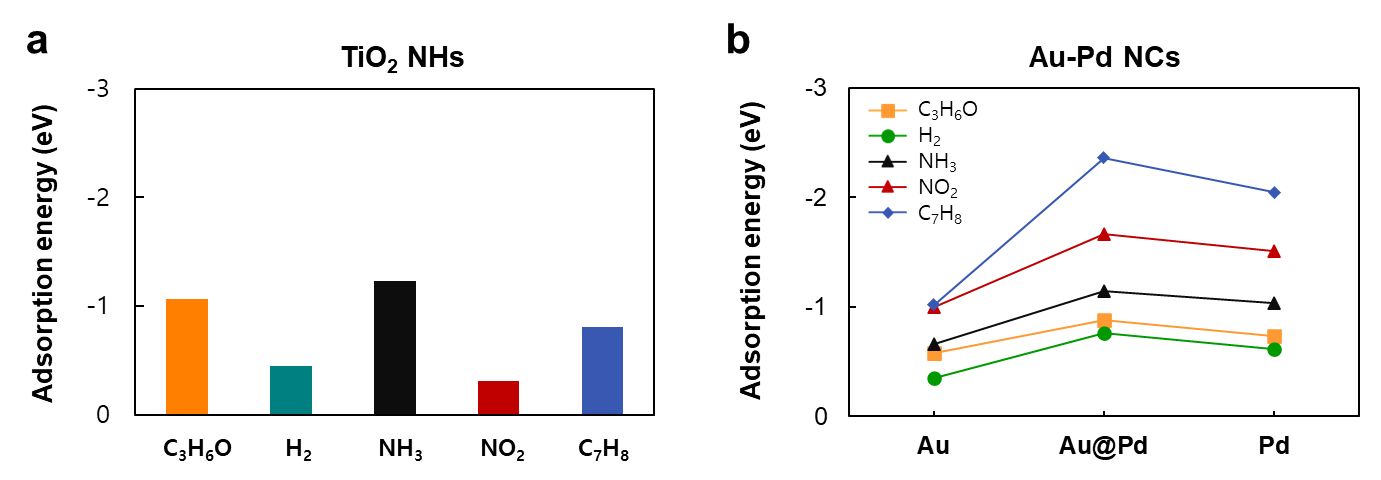


**figure S9.** Calculated adsorption energies of C_3_H_6_O, H_2_, NH_3_, NO_2_ and C_7_H_8_ on the surfaces of (a) TiO_2_ NHs, (b) Au, Pd, and Au@Pd NCs.


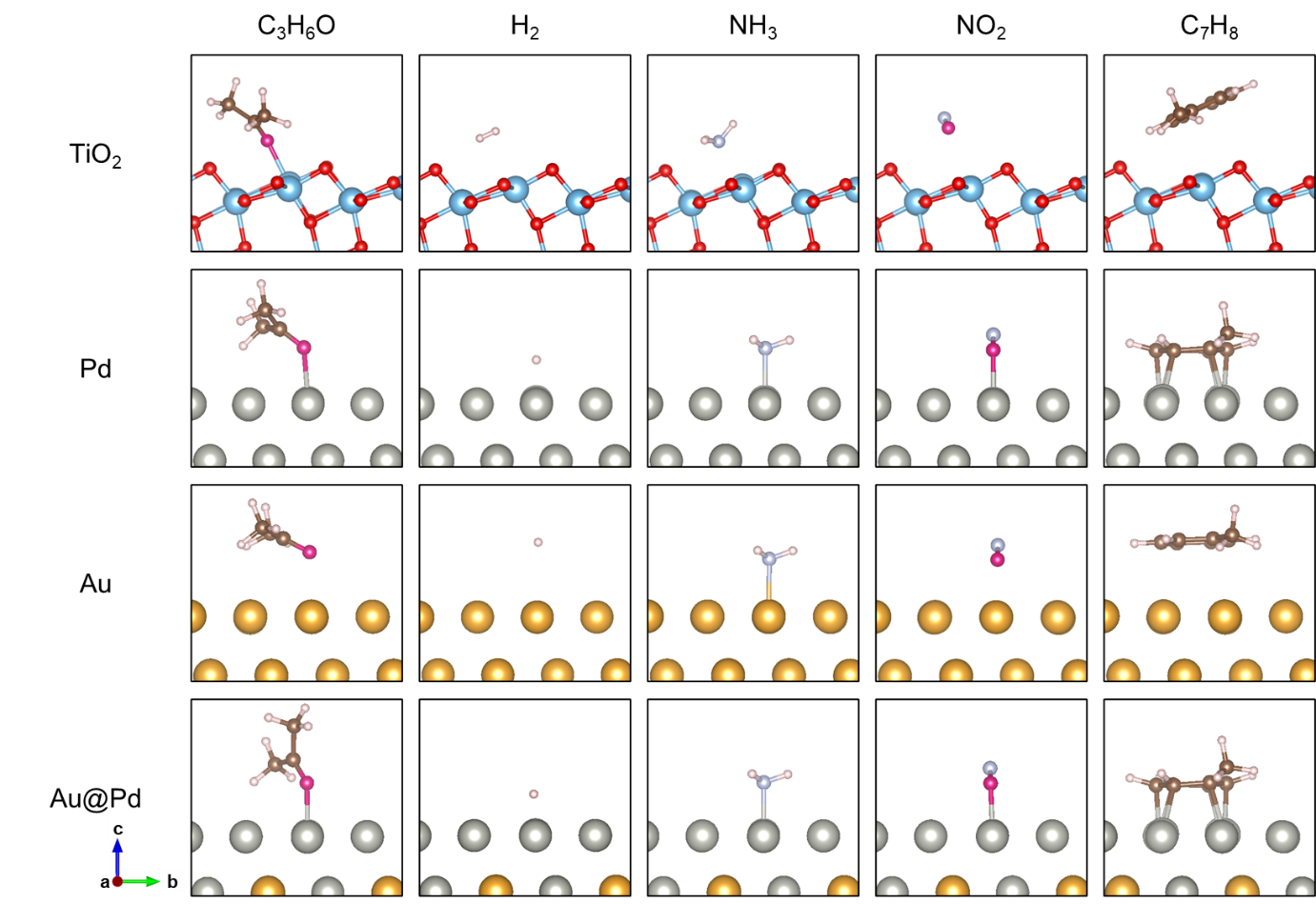


**Figure S10.** Side view of optimized adsorption structures for C_3_H_6_O, H_2_, NH_3_, NO_2_ and C_7_H_8_ on TiO_2_, Pd NCs , Au NCs, Au@Pd NCs surfaces.


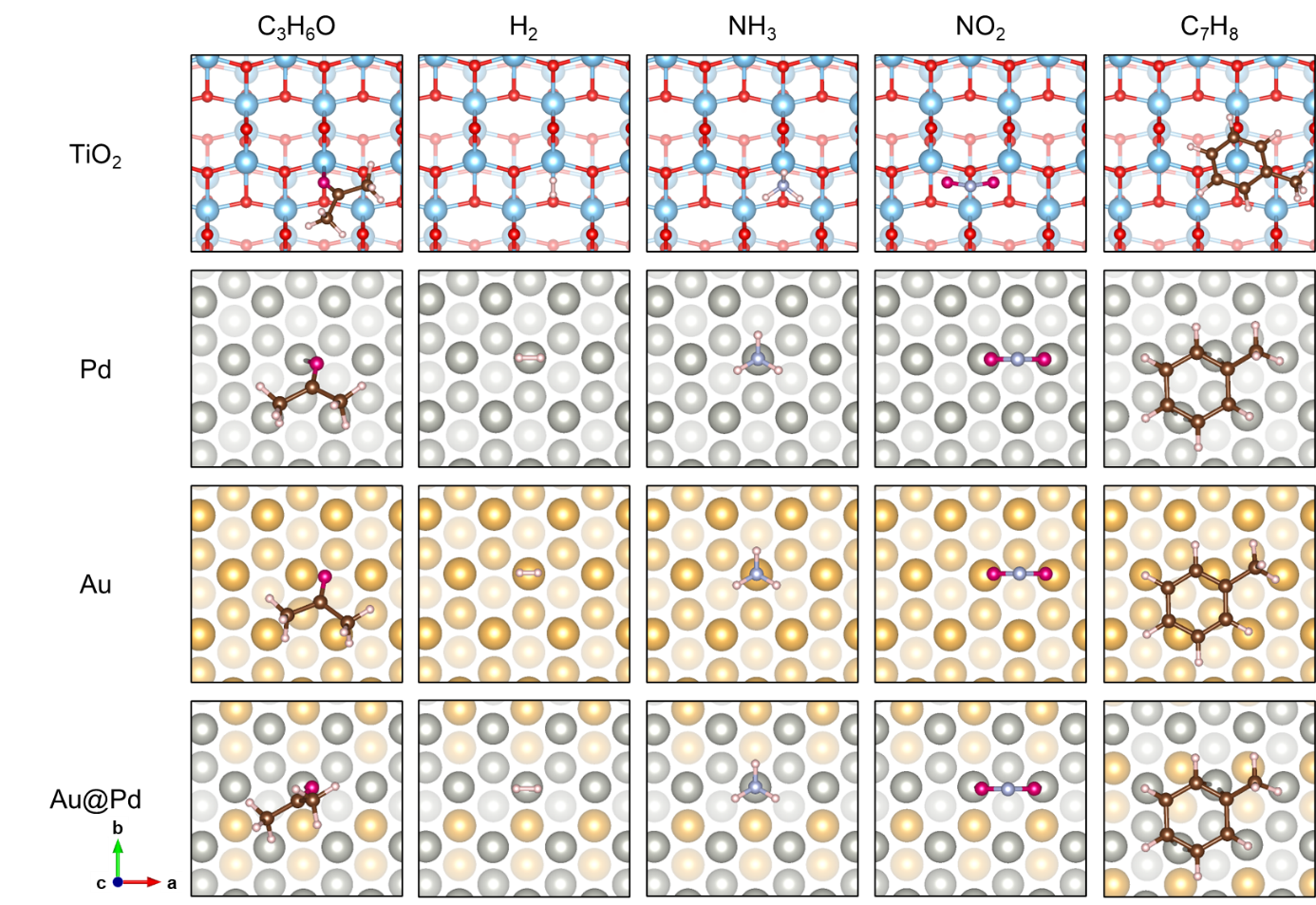


**Figure S11.** Top views of optimized adsorption structures for C_3_H_6_O, H_2_, NH_3_, NO_2_ and C_7_H_8_ on TiO_2_, Pd NCs, Au NCs, Au@Pd NCs surfaces.


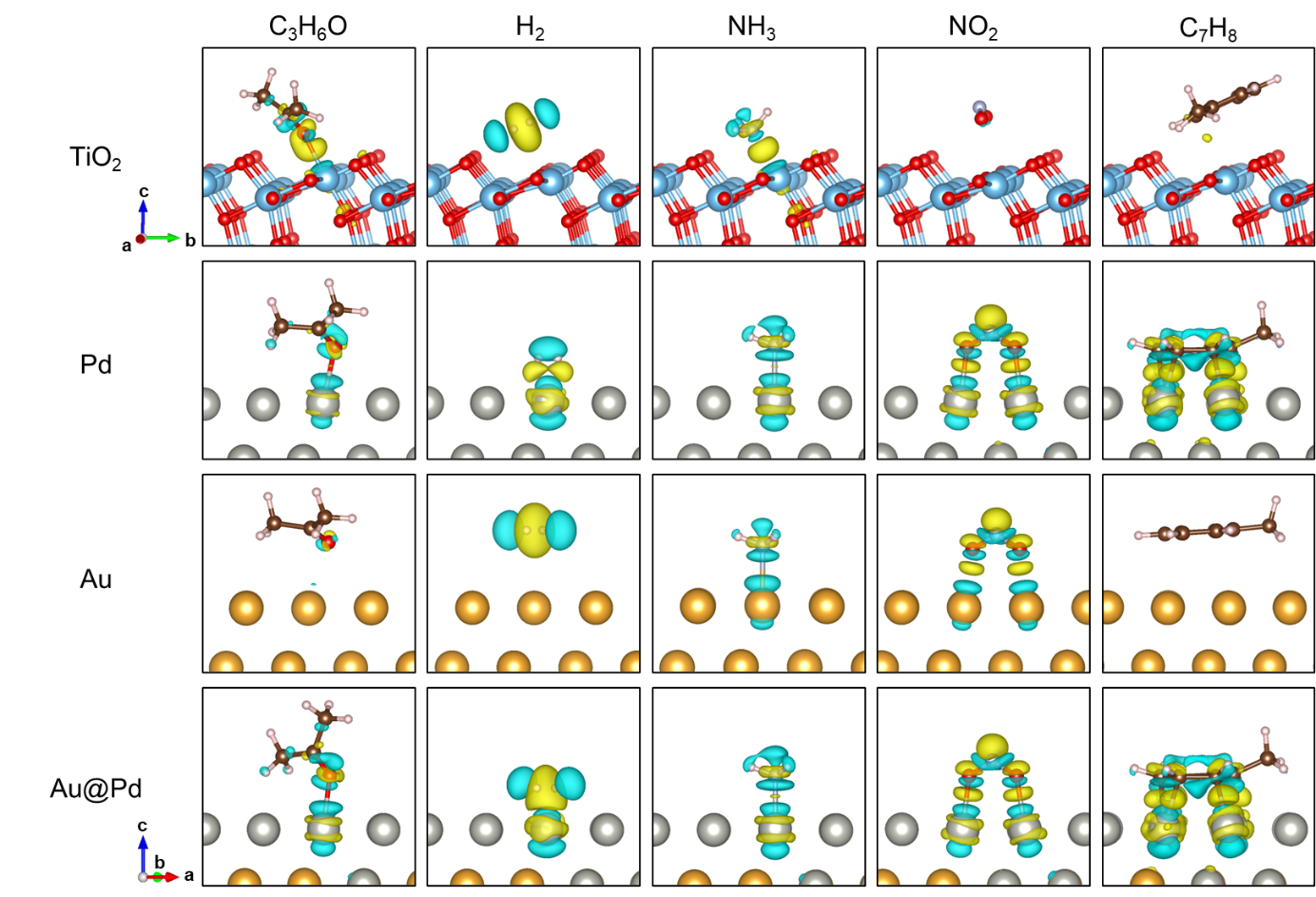


**Figure S12.** Charge transfer between adsorbates and TiO_2_, Pd NCs, Au NCs and Au@Pd NCs surfaces. Cyan region is electron-deficient and yellow region is electron-rich. The isosurface value is 0.004 e Å^-3^

**Table S1.** The blank signal of the Au@Pd NCs on TiO_2_ NHs gas sensor under 5 V applied at 200 °C (n=30)

| Blank | Response (R_a_/R_g_) | Blank | Response (R_a_/R_g_) | Blank | Response (R_a_/R_g_) |
| --- | --- | --- | --- | --- | --- |
| **1** | 1.009107 | **11** | 1.005387 | **21** | 0.993813 |
| **2** | 1.008050 | **12** | 1.003938 | **22** | 0.992973 |
| **3** | 1.005716 | **13** | 0.998708 | **23** | 0.997587 |
| **4** | 1.005810 | **14** | 1.001556 | **24** | 1.000698 |
| **5** | 1.000819 | **15** | 0.994689 | **25** | 0.993053 |
| **6** | 1.001623 | **16** | 0.991992 | **26** | 0.998915 |
| **7** | 1.001396 | **17** | 0.994522 | **27** | 1.001371 |
| **8** | 1.006229 | **18** | 0.996512 | **28** | 1.001088 |
| **9** | 1.001794 | **19** | 0.992180 | **29** | 1.003647 |
| **10** | 1.000691 | **20** | 0.996434 | **30** | 1.002250 |

**Table S2.** The lattice constants and d-band centers of surface models of Au NCs, Au@Pd NCs, tensile-strained Pd NCs, and Pd NCs. The Pd NCs model with an expanded lattice exhibited a higher d-band center compared to the unstrained Pd.

| model | | lattice constant (Å) | d-band center (eV) |
| --- | --- | --- | --- |
| 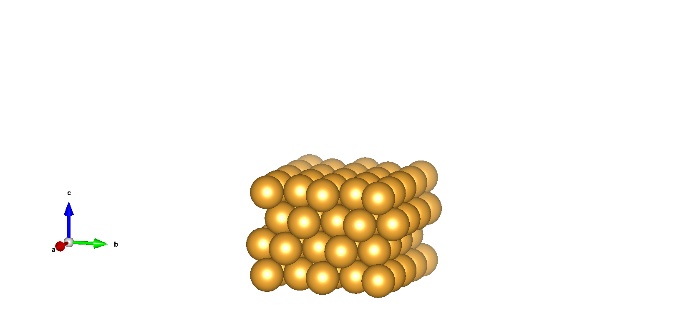 | Au NCs | 4.098 | -3.041 |
| 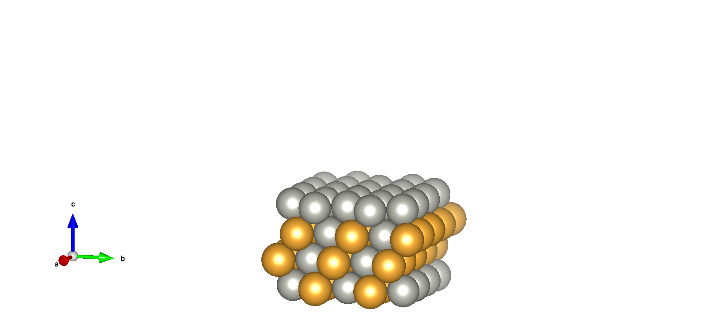 | Au@Pd NCs | 3.982 | -1.308 |
| 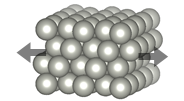 | tensile-strained Pd NCs (artificial) |  | -1.472 |
| 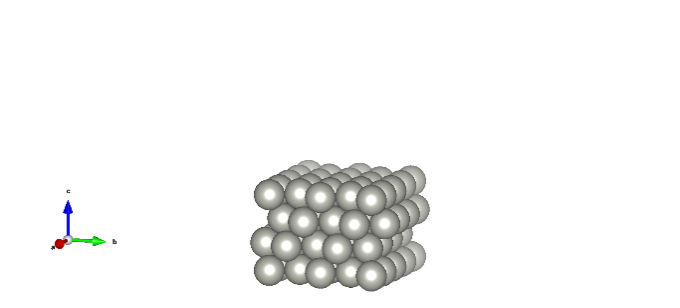 | Pd NCs | 3.886 | -1.621 |
